# Thermocouple and Infrared Sensor-Based Measurement of Temperature Distribution in Metal Cutting

**DOI:** 10.3390/s150101274

**Published:** 2015-01-12

**Authors:** Abdil Kus, Yahya Isik, M. Cemal Cakir, Salih Coşkun, Kadir Özdemir

**Affiliations:** 1 Vocational School of Technical Science, Uludag University, 16059 Bursa, Turkey; E-Mails: yyisik@gmail.com (Y.I.); coskuns@uludag.edu.tr (S.C.); 2 Department of Mechanical Engineering, Uludag University, 16059 Bursa, Turkey; E-Mails: mcemalcakir@gmail.com (M.C.C.); kadirzdemir@gmail.com (K.Ö.)

**Keywords:** tool temperature, thermocouple, machining, cutting speed, pyrometer

## Abstract

In metal cutting, the magnitude of the temperature at the tool-chip interface is a function of the cutting parameters. This temperature directly affects production; therefore, increased research on the role of cutting temperatures can lead to improved machining operations. In this study, tool temperature was estimated by simultaneous temperature measurement employing both a K-type thermocouple and an infrared radiation (IR) pyrometer to measure the tool-chip interface temperature. Due to the complexity of the machining processes, the integration of different measuring techniques was necessary in order to obtain consistent temperature data. The thermal analysis results were compared via the ANSYS finite element method. Experiments were carried out in dry machining using workpiece material of AISI 4140 alloy steel that was heat treated by an induction process to a hardness of 50 HRC. A PVD TiAlN-TiN-coated WNVG 080404-IC907 carbide insert was used during the turning process. The results showed that with increasing cutting speed, feed rate and depth of cut, the tool temperature increased; the cutting speed was found to be the most effective parameter in assessing the temperature rise. The heat distribution of the cutting tool, tool-chip interface and workpiece provided effective and useful data for the optimization of selected cutting parameters during orthogonal machining.

## 1. Introduction

During metal cutting, the magnitude of the temperature on the cutting edge is a function of the cutting parameters. This temperature directly affects the production. Heat generated during machining is affected by many events, including tool life, chip formation, surface quality, cutting forces, *etc.* Maximum heat occurs at the tool-chip interface. Thus, machinability can be improved by monitoring the cutting temperature of the tool.

Temperature estimation is one of the most difficult and complicated procedures in metal cutting operations. Due to the complexity of the various events taking place at the point of contact between the tool and workpiece, developing a model for measuring the temperature is an extremely difficult process. Consequently, accurate and repeatable temperature prediction still remains a challenge due to this complexity of the contact phenomena [1]. It is quite difficult to measure the temperature because the heat in the region is very close to the cutting edge. Due to the lack of sufficient experimental data, it is not possible to verify a mathematical model; however, numerous attempts have been made to measure the temperature in machining operations [2].

Lowen and Shaw [3] developed an analytical prediction model for the measurement of cutting temperatures during machining. They concluded that the cutting temperature is the function of cutting speed and feed rate. They specified the average cutting speed as:

(1) $$Ѳ\text{t}=V^{0.5}f^{0.3}$$

where Ѳt is the average cutting temperature, *V* is the cutting speed and *f* is the feed rate.

Temperature measurement techniques include thermocoupling with an embedded tool-chip pair, measurement of infrared radiation (pyrometers, infrared photography, *etc.*) and the use of thermo-sensitive paint, as well as metallography based on metal microstructure or microhardness variations, measurement of temper colour, and the use of thermal cameras. Each technique has its own advantages and limitations depending on the physical measurement [4–7].

Thermocouples are one of the most widely used experimental methods for measuring the temperature in machining. Thermocouples are conductive, inexpensive, can be operated over a wide temperature range and can be easily applied; however, they only measures the mean temperature over the entire contact area of the tool and the workpiece. Stephenson stated that, based on this measurement technique using thermocouples, the average electromotor force (emf) is in the tool-workpiece interface [8]. Grzesik [9] investigated the influence of the tool-work interface temperature when machining AISI 1045 and AISI 304 using coated tools. A standard K-type thermocouple inserted into the workpiece was used to measure the interface temperature. The friction on the flank face had a great influence on the heat generated at a cutting speed of approximately 200 m/min. Cotterm [10] measured the machined surface temperatures with two thermocouples inserted into the workpiece when machining aluminum 6082-T6. The results indicated that an increase in the cutting speed led to a decrease in the cutting forces and the machined surface temperatures. This reduction in the temperature was attributed to the higher metal removal rate that resulted in more heat being carried away by the chip.

Ay and Yang [11] used a technique with K-type thermocouples to analyze the temperature variations in carbide inserts when cutting various materials such as copper, cast iron, aluminum 6061 and AISI 1045 steel. They observed that oscillations in the temperatures at locations near the cutting edge were more rigorous for the ductile materials and less so in the hard-machining materials. Yujing *et al.* [12] reported an experimental investigation of the influence of cutting parameters on cutting temperature in the milling of Ti6AI4V by applying a semi-artificial thermocouple. Analysis results showed that the tool and workpiece temperature performed in a similar rising trend with the increase of cutting parameters including cutting speed, feed rate, radial feed, and axial feed, and that their degrees of influence decreased successively. Krishna *et al.* [13] investigated temperature prediction in the orthogonal machining of AI/SiCp composites. Using a thermocouple, experiments were conducted to measure the temperature along the cutting tool edge at various cutting speeds and depths of cut, keeping the feed rate constant, while turning with a K-20 carbide cutting tool. An analysis of the steady state heat transfer was carried out and the temperature distribution at the cutting edge, shear zone, and interface regions was reported.

In addition to thermocouples, IR techniques are probably the second most common method for measuring temperatures in machining. In the IR technique, the surface temperature of the body is measured based on its emitted thermal energy [14]. In this technique, the radiation from the tool, the workpiece and the chip is measured to establish the temperature on the outside surfaces of these regions [15]. Radiation techniques are contactless methods for measuring the surface temperature of a body based on its emitted thermal energy. This technique is possibly the most suitable in turning, where high temperatures can be easily captured, as there is no direct contact with the heat source. On the other hand, chip obstruction makes it difficult to measure the temperature at the tool-chip interface [2]. Lin *et al.* [16] used an infrared pyrometer to measure the tool-chip temperature for carbide and ceramic tools at a cutting speed of 600 m/min. Dewes *et al.* [17] employed an IR camera and the thermocouple technique to measure the chip temperature when machining H13 hardened steel in high-speed machining. In a study to compare the heat flow through brazed and bonded tools, an IR camera was used by Darwish *et al.* [18] to measure the tool-chip interface temperature in orthogonal cutting tests. Young [19] used an IR camera to measure the temperature of the chip back section and the interface temperature in the orthogonal cutting of AISI 1045 steel.

The influence of cutting parameters on tool temperature has been examined in other studies such as that of Chu and Wallbank in 1998, where a relationship was established between the cutting temperature and the cutting parameters for a specific range of cutting speeds and feed rates. The relationship of the workpiece temperature with the cutting parameters was established and the results showed that the temperature correlated well with the cutting speed and the feed rate, but had little effect on the nose radius [20]. Liang *et al.* [21] developed a three-dimensional inverse heat conduction procedure that was proposed to quantitatively determine the steady-state tool-chip interface temperature in dry turning. The infrared method was utilized to measure the temperature on the rake face of the insert during the transient cooling process after the feed motion was halted. With the experimentally measured temperature data, the 3-D heat conduction model of the cutter and an optimization scheme were used to solve the effective heat flux on the tool-chip interface and the interface temperature during turning. Zgorniak and Grdulska [22] measured the temperature distribution in the cutting zone during the end milling operation of magnesium alloy AZ91HP by employing the IR measurement technique. In summary, it is possible to observe temperature distribution and even monitor the temperature of chips created during a manufacturing process; however, the measurement of the temperature in machining is still a challenge that requires unique solutions.

In this study, the most widely used embedded K-type thermocouple and an infrared pyrometer were selected for the measuring of tool and the tool-chip interface temperatures during orthogonal metal cutting. The effects of the cutting speed, the feed rate and the depth of cut on the temperatures were observed. The heat distribution in the cutting tool, tool-chip interface and workpiece provided useful information about the optimization of selected cutting parameters.

## 2. Temperatures in Metal Cutting

In the cutting process, nearly all of the energy dissipated in plastic deformation is converted into heat that, in turn, raises the temperature in the cutting zone. Because heat generation is closely related to plastic deformation and friction, three main sources of heat can be specified when cutting: (1) plastic deformation by shearing in the primary shear zone; (2) friction on the cutting face; and (3) friction between the chip and the tool on the tool flank.

**Figure 1.**
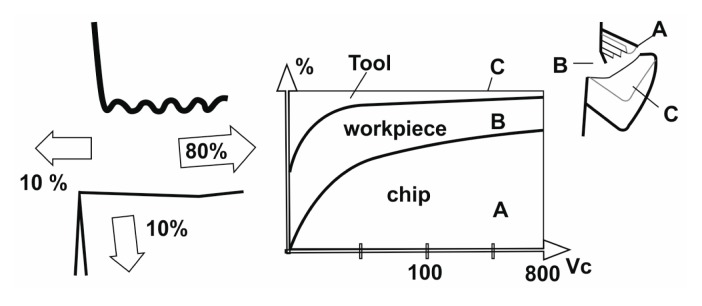
The balance of heat generation and heat dissipation in metal cutting [23].

Temperature causes dimensional errors on the machined surface. The cutting tool elongates as a result of the increased temperature, and the position of the cutting tool edge shifts toward the machined surface, resulting in a dimensional error of approximately 0.01–0.02 mm. Because the processes of thermal generation, dissipation, and solid body thermal deformation are all transient, some time is required to achieve a steady-state condition. Heat is mostly dissipated by the discarded chip, which carries away approximately 60%–80% of the total heat. The workpiece acts as a heat sink drawing 10%–20% of the heat away, and the cutting tool draws away ∼10% of the heat. The balance of the heat generation and the heat dissipation in metal cutting is shown in Figure 1.

**Figure 2.**
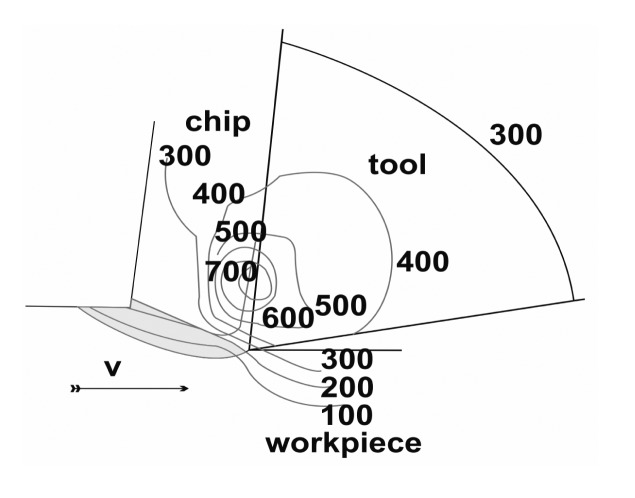
Co-temperature curves [24].

The cutting temperature is not constant throughout the tool, the chip or the workpiece. It can be observed that the maximum temperature is developed not on the very cutting edge, but at the tool rake, some distance away from the cutting edge. The temperature field in the cutting zones is shown in Figure 2.

The heat generated during the metal cutting process is due to the plastic deformation energy that transforms itself into the form of heat. The heat generation rate, Q (W), is given by Sata and Takeuchi [25,26] as:

(2) $$Q=1.68af^{0.15}V^{0.85}$$

where *a* is the depth of cut (mm), *f* is the feed rate (mm/rev), and *V* is the cutting speed (m/min).

## 3. Experimental Conditions

**Figure 3.**
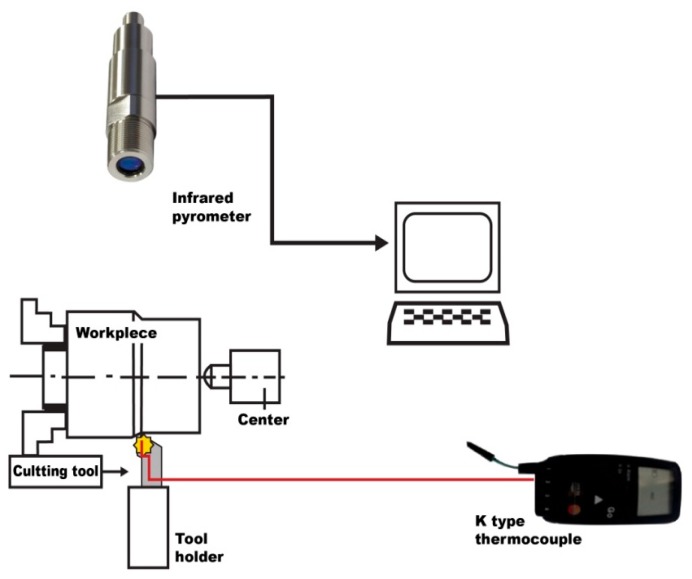
Schematic view of the experimental setup.

In this study, two methods of tool temperature evaluation are presented: the placement of a K-type thermocouple on the tool and the use of an infrared pyrometer. A schematic view of the experimental setup is shown in Figure 3 and experimental conditions are recorded in Table 1. The machining process was performed using a NR 2020K-08 tool holder and a PVD TiAlN-TiN-coated WNVG 080404-IC907 carbide insert. The workpiece material was an AISI 4140 alloy steel. The chemical composition of the workpiece material is shown in Table 2.

Cylindrical workpieces (Ø45 × 300 mm) were fixed between the chuck and the tailstock and were pre-machined using a separate insert. The samples were heat treated by induction hardening and a hardness of 50 HRC was maintained. The samples were then solution heat treated and oil quenched to achieve proper hardness. After the heat treatment, the material was normalized.

### 3.1. Infrared Pyrometer

The radiation technique uses contactless methods to measure the surface temperature of the body based on its emitted thermal energy. This technique is likely the most suitable in turning where high temperatures can be captured easily as there is no direct contact with the heat source. During the experiments, the tool-chip interface temperature was measured using an Optris CF4 infrared pyrometer, the specifications of which are shown in Table 3. To measure the temperature, the infrared pyrometer was placed precisely 45 cm above the tool rake face. Five signals were applied per second for the various cutting parameters. The size of the measurement spot was 1.5 mm, which corresponded to the distance to the measurement surface of 0.6 mm. The pyrometer had a response time of 200 ms. The selected measurement site was on the insert rake face at 1 mm from the cutting edge and just beside the leading edge in order to avoid chip obstruction. With the experimental apparatus for tool-chip interface temperature measurements using the infrared system with an IR (infrared radiation) pyrometer, the target image temperature ranged from 385 to 1600 °C. The minimum resolvable temperature difference was approximately ± 2° or ± 0.2%. System accuracy (at an ambient temperature of 23 ± 5 °C) was ± (0.3% of reading + 2 °C). The IR pyrometer was presumed to be more suitable as it is more accurate for use in turning processes where high temperatures can be captured easily as there is no directed contact with the heat source.

It is obvious that the maximum temperature zone was somewhat distant from the cutting edge, which theoretically corresponds to the zone of maximum wear of the tool, *i.e.*, the zone of maximal tool wear coincides with the maximum temperature zone. The contact area on the rake face is usually defined due to the fact that there is no universal method of measuring and predicting the area of contact between the tool and the chip. The contact area is in most cases controlled by the length of contact. The width of the region of contact is usually about the same size as the depth of cut.

#### 3.1.1. Construction and Operation of the Infrared Pyrometer

**Figure 4.**
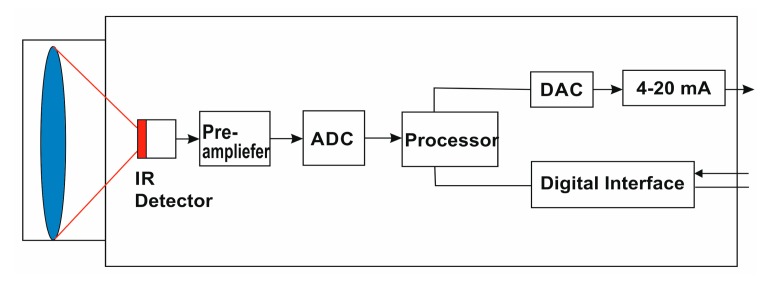
Block diagram of an IR pyrometer.

A block diagram showing the general construction of an infrared pyrometer can be seen in Figure 4. By use of input optics, the emitted object radiation was focused onto an infrared detector. The detector generates a corresponding electrical signal which is then amplified and may be used for further processing. Digital signal processing transforms the signal into an output value proportional to the object temperature. The temperature result is shown on a display. Consequently, the temperature of the measured object is mainly generated in three steps: (1) transformation of the received infrared radiation into an electrical signal; (2) compensation of background radiation from pyrometer and object; and (3) linearization and output of temperature information [27].

#### 3.1.2. Calibration of the Infrared Pyrometer

**Figure 5.**
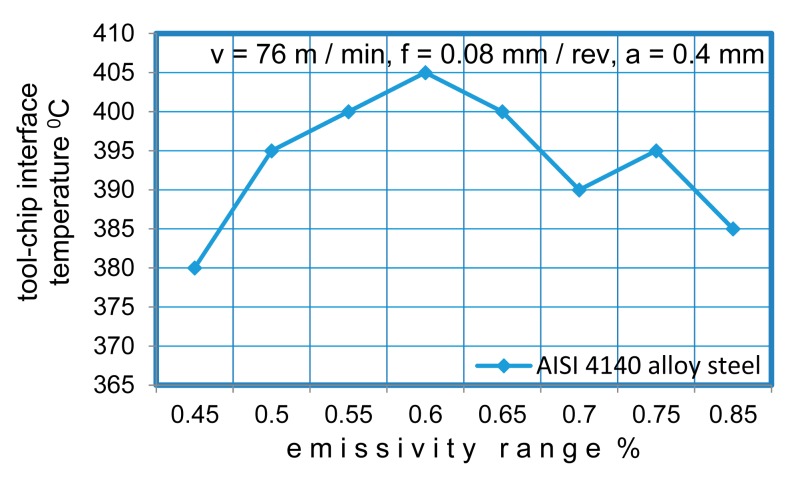
Emissivity range of the IR pyrometer.

Emissivity is the measure of the ability of an object to absorb, transmit and emit infrared energy. Emitted energy indicates the temperature of the object. It can have values ranging from 0 (shiny mirror) to 1.0 (black body). If a value of emissivity is higher than the actual set value, the output will read low, provided that the target temperature is above the ambient one. The emissivity of the material is the crucial parameter for thermal radiative heat transfer. The emissivities of an AISI 4140 alloy steel were measured as a function of the surface temperature. In the literature, the recommended emissivity value for AISI 4140 alloy steel is between 0.50 and 0.75 [28]. For an accurate measurement of the cutting temperature, different emissive values (in the range of 0.45–0.85) were tried in order to adjust the accuracy of the temperatures at constant cutting parameters. Figure 5 shows the temperature measurements at various emissivity values. The standard procedure was applied during the experiments to ensure reliable and repeatable results. The infrared pyrometer was calibrated as a standard device prior to its use to determine the transfer factors. To machine AISI 4140 alloy steel using TiAIN-TiN-coated tungsten carbide, the emissivity value of 0.60 was identified for a range of 385 °C to 600 °C.

### 3.2. The Thermocouple

**Figure 6.**
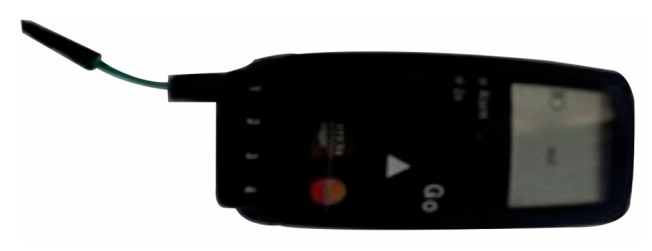
K-Type thermocouple display.

**Figure 7.**
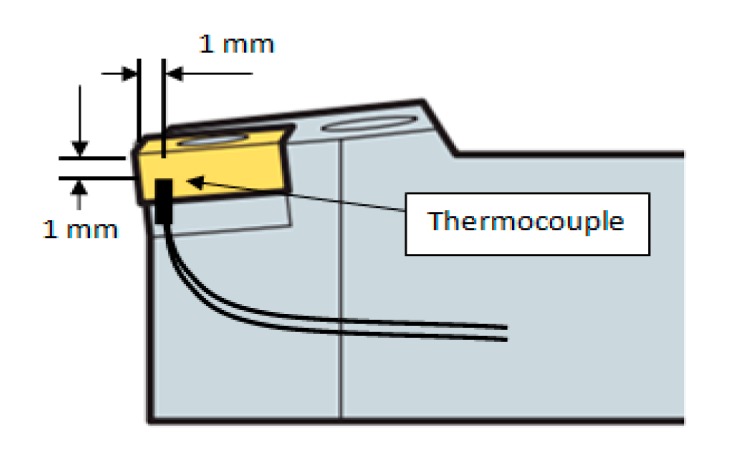
Position of thermocouple.

**Figure 8.**
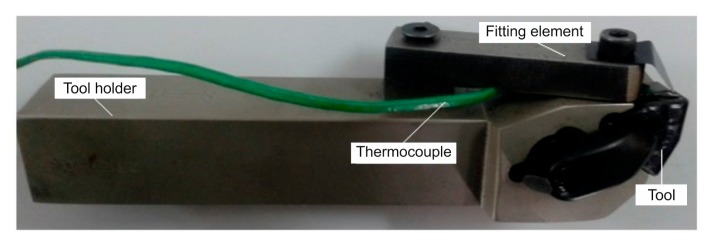
Mounting of thermocouple onto the tool holder.

The principle of temperature measurement by a thermocouple is that when two different metals come in contact, if these parts, called the hot and the cold junctions, are maintained at two different temperatures, an electromotive force (emf) is produced across these two junctions. The emf generated is a function of the materials used for the thermocouple as well as the temperatures of the junctions. In machining applications, a thermoelectric emf is generated between the tool and the workpiece [29]. For this study, the tool temperature was measured using a K-type thermocouple. In Figure 6, a K-Type thermocouple display is presented. The highest cutting temperature on the insert was observed 1 mm below the cutting edge. Therefore, the thermocouple set was mounted 1 mm away from the front surface and 1 mm from the upper surface of the cutting edge. Figure 7 displays the position of the thermocouple. Considering the depth of cut, there were some difficulties in mounting the thermocouple. These difficulties occurred because the tool-chip contact point varies continuously during cutting as a result of the variations in the cutting speed, the feed rate and the depth of cut. The thermocouple diameter was 0.5 mm and the temperature measurement range was −195 °C to 1100 °C. The mounting of a thermocouple is shown in Figure 8. For the study, the accuracy of the tool temperature measurements using the K-type thermocouple data acquisition system was ±2.5 °C or ±1%.

**Figure 9.**
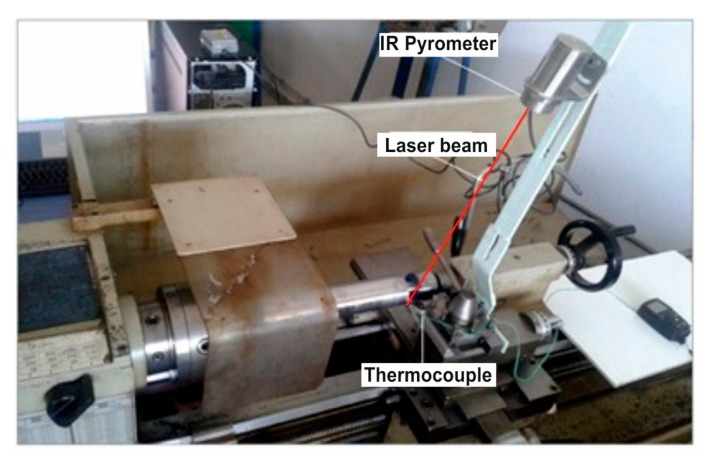
Thermocouple and IR pyrometer connections onto the lathe.

For the tool temperature measurements, a thermocouple with a TESTO 177 thermometer having four temperature inputs was used. In Figure 9, the thermocouple and the IR pyrometer connections on the tool holder are shown.

## 4. Methods

### 4.1. Experimental Procedure

The Taguchi L18 (21 × 37) method was used for the determination of optimum control factors. Low, medium and high cutting parameters were selected for the experiments. In the selection of these parameters, the tool manufacturers' recommendations and the machine tool capacity were taken into consideration. There were 18 (3 × 3 × 2) combinations of the turning tests in total which were all carried out to complete the experiments. The experimental conditions are shown in Table 4.

### 4.2. IR Pyrometer Measurement of Tool-Chip Interface Temperature

In this study, an infrared pyrometer, the Optris CF4, was used to measure the tool-chip interface temperature. The maximum temperature (approximately 525 °C) was recorded around the cutting zone. A total of 18 trials were conducted throughout these experiments and brand new inserts were used for each temperature measurement. Hence, the cutting temperature increased as the cutting speed, the feed rate and the depth of cut increased. During the experiments, in order to measure the tool-chip interface temperatures with the IR pyrometer, five measurements were taken per second and the measurements were averaged for each of the cutting parameters. The experiments were repeated three times. During the measurement process, because the cutting zone was very small and obstructed by chips, some difficulties were encountered due to occasional blockage.

### 4.3. Thermocouple Measurement of Tool Temperature

In the measurement of the temperature with the thermocouple, some difficulties were encountered in the region of the assembly closest to the cutting zones. To avoid this situation, a special apparatus was designed and manufactured for the thermocouple connection to the tool holder. The thermocouple measurements were recorded every five seconds. The measurements were repeated three times for the same cutting parameters and the measurement results were averaged.

## 5. Analysis of Experimental Results

This paper presents a combination of two techniques of temperature measurement used simultaneously in order to evaluate the temperature distribution in metal cutting operations. It was observed that the application of simultaneous measurements (both chip temperature and tool temperature) in machining is unique and there are very few related works encountered in the literature.

Not much information is to be found in the literature dealing with heat distribution on the chip, tool and workpiece. Only a few ratios are given and these ratios are highly dependent on cutting parameters. There are not many works dealing with the influence of cutting speed and its association with heat distribution.

Some practical difficulties are also discussed in this paper. The difficulty of direct measurement of localized high temperatures at the contact interface between the cutting tool and the sliding chip occurred because the site was constantly obscured throughout the cutting. Chip obstruction made it difficult to measure the temperature.

The use of thermocouples to measure the tool-chip interface temperature is not possible due to the blocking of the cutting chips and the difficulty of mounting the thermocouples precisely to the shear zone. Therefore, an IR pyrometer is required to measure the tool-chip interface temperature.

In the metal cutting industry, heat is generally dissipated by the discarded chip, which carries away approximately 70%–80% of the total heat. The workpiece acts as a heat sink drawing 10%–20% of the heat away and the cutting tool draws away ∼10% of the heat.

The heat distribution of the cutting tool, tool-chip interface and workpiece can provide useful information about the optimization of selected cutting parameters. Therefore, the relationship between the cutting parameters and heat distribution needs to be precisely determined. The aim of the present paper was to establish this relationship. Simultaneous temperature measurements were made during the experiments and the experimental results are given in Table 5.

During the machining process, there are many advantages to using the multi-sensor approach as measuring the tool and tool-chip interface temperatures. This method can provide extremely useful information as to whether or not the optimal cutting parameters have been selected by determining the ratio of the cutting tool temperature to the tool-chip interface temperature. Moreover, this method makes it possible to determine the optimum cutting parameters.

### Effect of Cutting Conditions on Temperature

In the experiments, the influence of the cutting parameters such as cutting speed, feed rate and depth of cut on the cutting temperature of AISI 4140 was observed. The higher the cutting speed, the higher the tool-chip interface temperature increase, resulting in a significant temperature difference.

**Figure 10.**
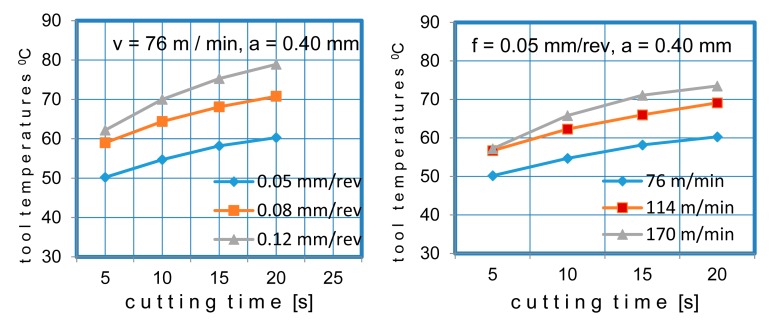
Change of tool temperature during the cutting process, depending on the time.

During the cutting process, an increase in the temperature was observed depending on the processing time. This was due to the tool wear and the increased friction at the cutting zone, which was taken into consideration in the tool temperature calculations. Figure 10 shows the variations of the tool temperature with the time at various cutting speeds and feed rates.

**Figure 11.**
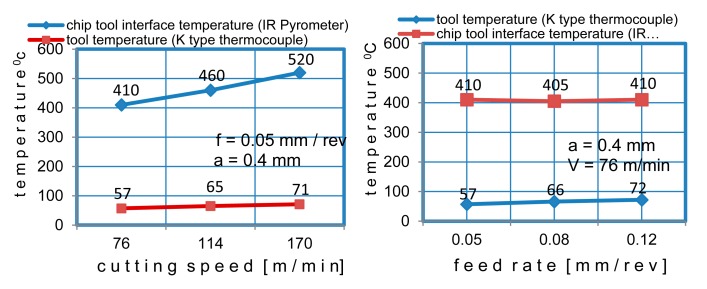
The influence of cutting speed and feed rate on temperatures.

The cutting speed is the cutting parameter that affects tool life most. However, there are some positive effects of increasing the cutting speed (such as improvement in friction). During the metal cutting, a large amount of the heat was removed from the workpiece by the chip. As the cutting speed gets higher, more heat is removed, but the tool temperature increases as well (see Figure 1). Reducing the cutting speed increases the amount of heat flowing to the workpiece, which causes the temperature of the workpiece to rise. Although increase in cutting speed increases the temperatures abruptly, the feed rate has very little effect on the temperature rise. The variations of tool temperature and the tool-chip interface temperature with the cutting parameters are shown in Figure 11. It can be readily observed that the tool-chip interface temperature and the tool temperature rise with the increase in cutting speed.

**Figure 12.**
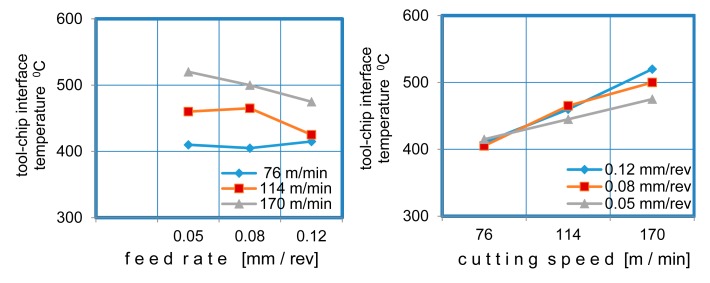
The influence of feed rate and cutting speed on tool-chip interface temperature.

During the experiments, PVD TiAlN-TiN-coated carbide inserts were used. The temperature increase in the tool is more limited compared to the increase in the tool-chip interface temperature due to the poor thermal conductivity of the coating material. The tool-chip temperature interface increase by 30% as the cutting speed increased; however, no significant temperature rise was observed for the feed rate. Both parameters had similar effects on the tool temperature. The influence of the feed rate and the cutting speed on the tool-chip interface temperature is shown in Figure 12.

**Figure 13.**
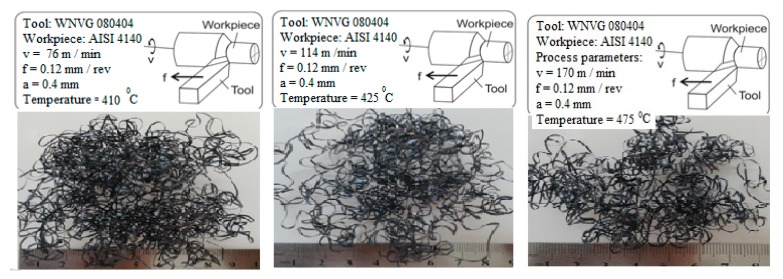
Chips form at different tool-chip interface temperatures and cutting speeds.

The tool-chip interface temperatures rose with the increase in cutting speed, and the chip colour became darker due to the increasing disposal rate of the chips. Significant changes in the form and the curvature of the chips were also observed. Figure 13 shows the chip forms at different tool-chip interface temperatures and different cutting speeds.

## 6. Finite Element Simulation of Cutting Temperature

Finite element analysis (FEA) is the most useful and accurate approach for the determination of field variables. This method is made possible by advancements in computing and computer processing powers and thus it has been used for almost all computer aided design methodologies in recent years. Applications range from deformation and stress analysis to field analysis of heat flux, fluid flow, magnetic flux, seepage and other flow problems. In this method of analysis, a complex region defining a continuum is disassembled into simple geometric shapes called finite elements.

Moreover, in order to verify the test results, it was essential to employ the FEM (finite element method). In this study, there was a good agreement between the test results and the FEM results. In this work, the application of FEM for thermal analysis of a single-point cutting tool in a turning operation is presented. Once the model for the determination of the temperature field for the single-point cutting tool was developed and verified using the experimental results, it was used to observe the effects of various parameters including the cutting parameters, the tool geometry, the tool material, the distance of the sensors from the cutting region and the rate of heat dissipation.

### Finite Element Model

A 3-D finite element simulation model was developed for simulation of the temperature based on the estimated heat over the tool-chip contact area. The real geometry of the cutting tool and the tool holder model were designed to achieve more realistic and accurate simulation results. The maximum temperature of 520 °C was recorded at the tool-chip interface. The heat converted from the work of plastic deformation and friction was assumed to be the same for the case in the simulations. The geometry and the material properties of the tool and the tool holder are listed in Table 6.

The convective heat transfer coefficient for the top surface of the cutting tool on the tool holder was set at 20 W/m^2^°C and applied to the top corner of the insert, where the heating zone on the corner was held constant: A = π × 1 mm^2^/4 = 0.78 × 10^−6^ m^2^. As for a typical cutting tool simulation, a heat flow (Q) of 17 W was used. The temperature near the heat source was observed to be 412 °C.

**Figure 14.**
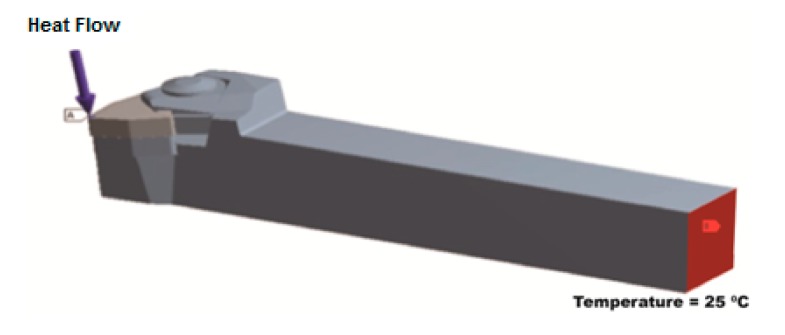
Boundary conditions.

**Figure 15.**
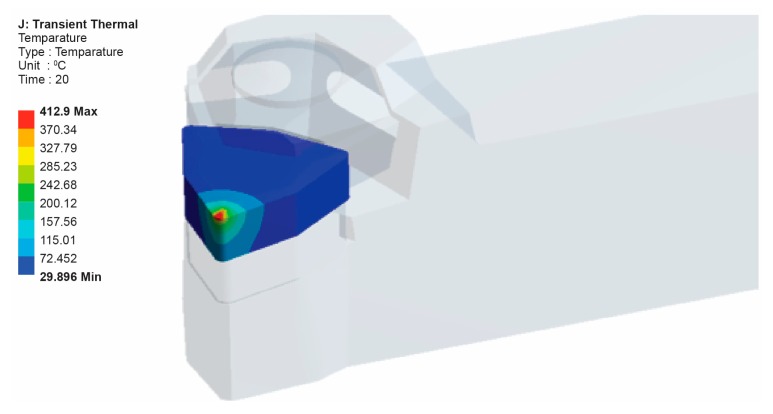
Temperature distribution on the cutting insert.

The thermal and the mechanical properties of the cutting insert and the tool holder material are given in Table 6. The transient thermal analysis was performed using an ANSYS commercial finite element code. The calculated heat flux applied to the tool-chip contact area on the rake face of the cutting insert was the basis of the thermal analysis. Figure 14 shows the tool-chip contact area on the cutting insert to which heat flux was applied. The boundary conditions applied on the finite element model can be seen in Figure 15. The calculated heat flow on the tool-chip interface was 17 W. Other surfaces, except for the end of the tool holder, were set to be adiabatic and the end of the tool holder was set to the ambient temperature. The ambient temperatures considered were in the range of 20 °C to 30 °C. The adiabatic boundary condition of the rest of the exposed surface was based on the boundary conditions set by Tay *et al.* [30].

**Figure 16.**
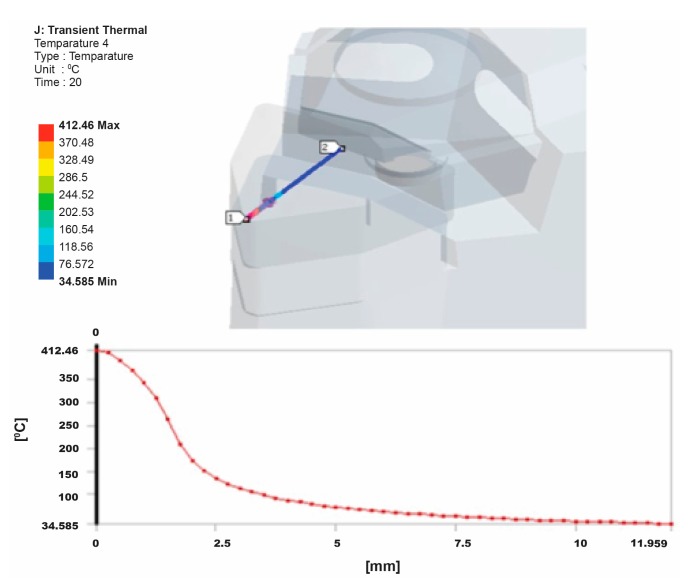
Temperature distribution on the path from the tool-chip interface.

It is possible to evaluate the heat distribution into the cutting tool by employing a FEM model, reducing the available heat flux until the simulated temperature matches with the experimentally measured temperature. Figure 16 shows the temperature distribution of between 29.89 °C and 412.9 °C on the tool-chip interface as well as the temperature distribution along the 12 mm path that starts from the tool-chip interface.

**Figure 17.**
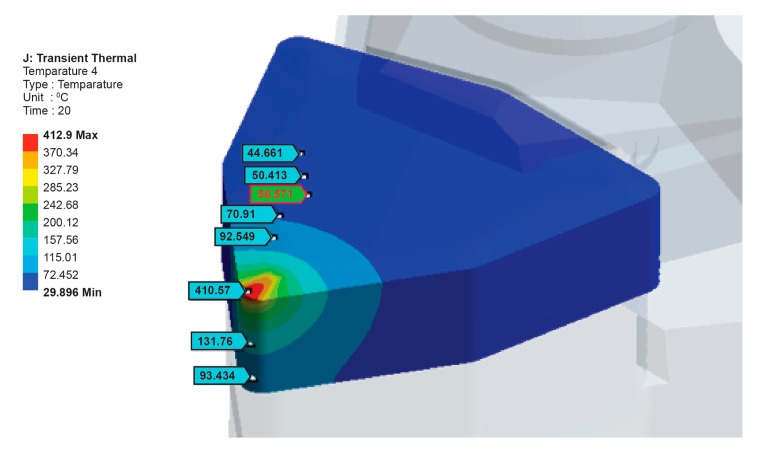
Temperature probe values for some locations.

The temperature results obtained from the experimental tests were 410 °C on the tool-chip interface and 57 °C near the tool-chip interface for a 0.4 mm cutting depth, a 76 m/min cutting velocity and a 0.05 mm/rev feed rate. For the verification and confirmation of the experimental results, the temperature distribution was simulated by using FEA. Comparison of the temperature values obtained by the finite element results and the experimental results showed good agreement. Figure 17 shows the temperature probe values for some locations.

## 7. Conclusions

In the present paper, techniques using an IR pyrometer and a K-type thermocouple were used simultaneously to measure the tool-chip interface and the tool temperature in turning heat-treated AISI 4140 alloy steel (hardness 50 HRC). This multi-sensor application in metal cutting provided information about the effects of cutting parameters on heat distribution. These applications (both chip temperature and tool temperature measurements) are unique in a machining context, and not many related works on the subject have been encountered in the literature. The heat distribution of the cutting tool, tool-chip interface and workpiece can provide useful information about the optimization of selected cutting parameters. Therefore, it is essential that the relationship between the cutting parameters and heat distribution be determined.

It was concluded that the cutting speed was the parameter most affecting the tool-chip interface temperature. The effect of the feed rate was not significant. Both parameters had similar effects on the tool temperature. Significant changes in the form and curvature of the chip were also observed as the cutting speed increased.

A 3-D finite element simulation model was also developed for the replication of the temperature based on the estimated heat over the tool-chip contact area. Comparison of the temperature values obtained by the finite element and the experimental results showed good agreement which means the FEM model of heat distribution was accurately constructed for use in future studies. The FEM analysis was essential in order to verify the test results.

## Figures and Tables

**Table 1.** Experimental conditions.

Machine tool	Harrison M300
Work specimen's materials	AISI 4140 alloy steel
Size	Cylindrical workpiecese Ø45 × 350 mm
Cutting tools	PVD coated carbide TiAIN-TiN cutting insert, WNVG 080404
Tool holder	DWLNR 2020K-08
Working tool geometry	Inclination angle:-6, rake angle:-6, clearance angle: 6, edge angle: 80
Principal nose radius	0.4 mm
Measurement temperatures	Optris CF4 infrared pyrometer
Thermocouple	K-type thermocouple, TESTO 177
Cutting speed, V	76 m/min, 114 m/min, 170 m/min
Feed rate, f	0.05 mm/rev, 0.08 mm/rev, 0.12 mm/rev
Depth of cut, a	0.40 mm, 0.60 mm
Environment	Dry

**Table 2.** Chemical composition of AISI 4140 alloy steel (vol%).

**C**	**Cr**	**Ni**	**Mn**	**P**	**S**	**Si**	**Mo**
0.38	0.80	9.58	0.75	0.035	0.04	0.15	0.15

**Table 3.** Specifications of the IR (infrared radiation) pyrometer.

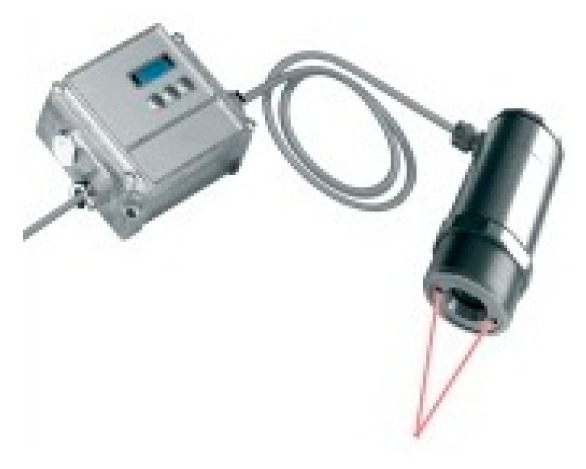	Temperature range	385 °C to 1600 °C (2 MH)
	Spectral range	1.6 μm
	Optical resolution (90% energy)	300:1 (2 MH)
	System accuracy (23 ± 5 °C)	±1% or ±1 °C2)
	Repeatability (23 ± 5 °C)	±0.3% or ±0.3 °C2)
	Temperature resolution	0.1 K
	Response time (90% signal)	10 ms
	Ambient temperature	−20 °C to 85 °C
	Storage temperature	−40 °C to 85 °C
	Optical parameter	1.5 mm @ 450 mm

**Table 4.** Machining parameters and their levels.

**Parameters**	**Levels**		

	**1**	**2**	**3**
Cutting speed *V*, (m/min)	76	114	170
Feed rate *f*, (mm/rev)	0.05	0.08	0.12
Depth of cut *a*, (mm)	0.40	0.60	

**Table 5.** Experimental results.

**No**	**A [mm]**	**V [m/min]**	**f [mm/rev]**	**Tool Temperature [°C] (Thermocouple)**	**Tool-Chip Interface Temperature [°C] (IR Pyrometer)**	**Tool-Chip Interface/Tool Temperature Percent Contribution (%)**
1	0.4	76	0.05	57	410	0.139
2	0.4	76	0.08	66	405	0.162
3	0.4	76	0.12	72	410	0.175
4	0.4	114	0.05	65	460	0.141
5	0.4	114	0.08	61	465	0.131
6	0.4	114	0.12	67	445	0.150
7	0.4	170	0.05	65	520	0.125
8	0.4	170	0.08	67	500	0.134
9	0.4	170	0.12	71	475	0.149
10	0.6	76	0.05	72	400	0.180
11	0.6	76	0.08	80	390	0.205
12	0.6	76	0.12	76	395	0.192
13	0.6	114	0.05	80	430	0.186
14	0.6	114	0.08	75	435	0.172
15	0.6	114	0.12	83	420	0.198
16	0.6	170	0.05	81	485	0.167
17	0.6	170	0.08	67	525	0.128
18	0.6	170	0.12	69	480	0.143

**Table 6.** Input values of the FEA (finite element analysis) model for the cutting tool.

Tool holder width and height	20.00 mm
Initial temperature	25 °C
Tool holder length	120.00 mm
Insert thickness	4.00 mm
Density of insert	14,900 kg/m^3^
Thermal conductivity of insert	36 W/m K
Specific heat of insert	302 J/kg K
Density of tool holder	7850 kg/m^3^
Thermal conductivity of tool holder	16 W/m K
Specific heat of tool holder	434 J/kg K
